# Women Quotas vs. Men Quotas in Academia: Students Perceive Favoring Women as Less Fair Than Favoring Men

**DOI:** 10.3389/fpsyg.2020.00700

**Published:** 2020-04-28

**Authors:** Miriam K. Zehnter, Erich Kirchler

**Affiliations:** Department of Applied Psychology, Work, Education and Economy, Faculty of Psychology, University of Vienna, Vienna, Austria

**Keywords:** women quotas, men quotas, preferential treatment, gender equality, support paradox, system justification, academia, free associations

## Abstract

In this study, we analyze the free verbal associations to the stimuli *women quotas* and *men quotas* of 327 medical students. Women and men quotas are characterized by the same *modus operandi* (i.e., preferential treatment based on sex/gender). However, women quotas help a low-status group, whereas men quotas help a high-status group. In line with a support paradox, that is, the perception that support for women is less fair and less legitimate than support for men, we expected that students would reject women quotas in academia more vehemently than men quotas. Specifically, we hypothesized that students would have more negative and more emotional associations with women quotas than men quotas. As predicted, students had more negative associations with women quotas than with men quotas. However, students did not have more emotional associations with women quotas than with men quotas. In addition, we explored the semantic content of the free associations to identify specific concerns over each quota. Students perceived women quotas as counterproductive, derogatory, and unfair, whereas they perceived men quotas as beneficial and fair. Concerns over the negative perceptions of quota beneficiaries were associated more frequently with women quotas than men quotas. Potential factors underlying students’ perceptions of both quotas are discussed.

## Introduction

In the 21st century, women represent a small proportion of political and economic leaders. In 2019, only 11 percent of elected head of states and 24 percent of parliamentarians worldwide were women (Catalyst, 2019a; UN Women, 2019). Moreover, women lead less than five percent of Fortune 500 companies and hold only 29 percent of senior management roles (Catalyst, 2019b). In academia in many countries (e.g., EU, U.S.), women hold half of the doctoral degrees, but represent only one-third of researchers (UNESCO Institute for Statistics, 2018; European Commission, 2019). Enhancing the opportunities for women, there has been an upsurge in the use of women quotas in politics and economics in the recent years (Bonitz, 2017; International Institute for Democracy and Electoral Assitance, 2018). Most prominently, the European Union pushed for mandatory women quotas in corporate boardrooms (Boffey, 2017; Zillman, 2017). In the US, California was the first state to introduce mandatory women quotas in corporate boards in 2018 (Ortiz, 2018).

In academia, the application of women quotas, when hiring research personnel, for the composition of evaluation boards, and for research grants and fellowships has also been discussed, and some countries (e.g., Austria^1^) have implemented them (Wallon et al., 2015). Women quotas are defined as an instrument aimed to accelerate the achievement of gender-balanced participation and representation by establishing a defined percentage of positions, which are allocated to women, generally under certain rules or criteria (European Institute for Gender Equality, 2019). Women quotas are widely debated and stir up controversy (He and Kaplan, 2017; Debating Europe, 2018). Their usefulness in increasing fairness and meritocracy has also been discussed within the scientific community (Seierstad, 2016; Terjesen and Sealy, 2016; Madison, 2019). In contrast, men quotas are discussed considerably less. Nonetheless, the idea of using men quotas in women-dominated academic fields (e.g., psychology) has gained popularity. Men quotas have been discussed for hiring research personnel (Kutter, 2013; Die Presse, 2014) and to increase the number of male students in currently women-dominated study fields, such as psychology and medicine (Lindstad, 2017; Dordowsky, 2018).

Women and men quotas are characterized by the same *modus operandi* (i.e., preferential treatment based on sex/gender^2^). However, women quotas and men quotas differ in one important way. On average, women compared to men appear to have lower social status, which is, among other factors, indicated by their lower political participation (Catalyst, 2019a; UN Women, 2019) and their employment in lower-status professions (Block et al., 2018; England, 2010; for indicators of social status see, Kawachi et al., 1999; Hollingshead, 2011). Moreover, empirical research showed that feminine attributes were associated with lower status than masculine attributes (Rudman et al., 2012). Lastly, Austria, where this study was conducted, is number four worldwide in valuing masculine attributes over feminine attributes (Hofstede et al., 2010). Thus, women quotas may help a low-status group, while men quotas may help a high-status group.

In the debate about women and men quotas in academia, the lay perceptions of students who may not have specific expertise and detailed knowledge of quotas have often been neglected. However, research has shown that lay conceptualizations of public policy (e.g., affirmative action, quotas) can be incorrect, but nevertheless influence reactions to it. For example, reactions to affirmative action were based on what people *thought* such measures were, rather than on what they actually were (Bell et al., 2000). The meaning ascribed to the term “affirmative action” predicted support for affirmative action (Golden et al., 2001). Lay conceptualizations did not only influence attitudes toward affirmative action, but also toward its beneficiaries (Arriola and Cole, 2001).

Accordingly, students’ thoughts about quotas may impact their views of (presumed) beneficiaries among research and teaching personnel, e.g., as competent mentors. In this manner, student’s perceptions of quotas may impact the effectiveness of quotas, such as the quotas’ capacity to provide female or male role models. For example, female role models only inspired motivation in younger women when their success was attributed to skill and effort, rather than luck or external help (McIntyre et al., 2011). Research showed that women who were associated with preferential treatment were indeed perceived to be less competent and less effective compared to women who were only selected based on merit (Heilman et al., 1992, 1998; Heilman, 1996; Resendez, 2002).

The aim of this study is to compare students’ spontaneous thoughts about women quotas and men quotas in academia. To this end, we examine students’ free verbal associations with both quotas, respectively, in terms of both valence and specific associations that may explain their evaluations. This may provide additional insights in regard to whether reactions to quotas are solely based on their *modus operandi* (i.e., preferential treatment based on sex/gender) or also on their target (i.e., women vs. men).

Free verbal associations provide a useful approach to assess students’ thoughts of quotas. In a free association task, research participants are presented with a stimulus word (sentence, picture, etc.) and asked to write down every association that comes spontaneously to their minds (Nelson et al., 2000). Unlike structured questions that can lead into a predetermined direction, free associations allow a great amount of freedom of expression (Gangl et al., 2012), and are thought to elicit default thinking (Rozin et al., 2002). Since free associations tap into people’s naturalistic thoughts and feelings, they are ecologically valid, and investigator interference is minimal (Joffe and Elsey, 2014).

### The Impact of Women Quotas vs. Men Quotas

In politics and business, the use of women quotas was associated with increased numbers of women holding political office (Meier, 2004; Tripp and Kang, 2008; Jones, 2009; Schwindt-Bayer, 2009; Paxton et al., 2010; Bonomi et al., 2013; Darhour and Dahlerup, 2013; Högström, 2016) and the proportions of women on company boards (Storvik and Teigen, 2010; Wang and Kelan, 2013; Sabatier, 2015). Evidence from a simulation study revealed that women quotas may be beneficial for all of society by encouraging highly qualified women to apply for high job positions, while discouraging mainly low qualified men from application (Stark and Hyll, 2014). In experimental studies, women quotas increased the likelihood of women entering competition (Balafoutas and Sutter, 2012; Niederle et al., 2013). The use of tie break quotas (i.e., the preferential treatment of women under the condition of equal qualification) increased women’s perceived organizational fit and their willingness to apply for a job, whereas the use of rigid quota regulations did not (Nater and Sczesny, 2016). Moreover, in competitive situations, quotas increased the performance of beneficiaries and non-beneficiaries (Calsamiglia et al., 2013). The analysis of companies’ disclosure statements of the skills of appointed board members revealed that women brought unique skill sets to corporate boards (Daehyun and Starks, 2016). A time series analysis of publicly available indicators of company performance suggests an association between greater diversity on boards, better organizational performance and more efficient risk taking and innovation processes (Bernile et al., 2018). A quasi-experiment in the political sphere showed that women quotas helped break down negative stereotypes about female politicians (De Paola et al., 2010).

On the downside, experimental studies showed that the use of women quotas decreased organizational attractiveness among potential applicants (Shaughnessy et al., 2016). Research experiments also showed that it was quotas’ perceived effectiveness that made organizations less attractive, in particular to male applicants (Windscheid et al., 2017). In Norway, where women quotas for corporate boards were mandated in 2006, the value of affected companies sank in the subsequent years, presumably because of the necessity to hire less experienced and younger women (Ahern and Dittmar, 2012). Other studies interpreted the decrease in short-term profits in Norwegian companies as a result of fewer workforce deductions, higher labor cost, and higher levels of employment, which may increase long-term profit (Matsa and Miller, 2013). The analysis of publicly available data on company performance further showed that greater gender diversity was beneficial for some companies, whereas it affected the value of others negatively (Adams and Ferreira, 2009). In particular, women quotas may negatively affect the value of small, young, profitable, and non-listed companies (Bøhren and Staubo, 2016). In addition, women quotas may not have the intended effects. Despite mandatory women quotas in Norway, female directors reported having less influence in decision making and feeling less as a part of the inner circle compared to male directors (Storvik and Gulbrandsen, 2016). In fact, women quotas may have negative consequences for women. Experimental research found that women who were associated with preferential treatment perceived themselves as less competent compared to women selected only based on merit (Heilman et al., 1987; Heilman, 1996; Unzueta et al., 2010). They were also perceived as less competent and effective by others (Heilman et al., 1992; Heilman and Welle, 2006). Accordingly, some have argued that the improvement of meritocratic assessment would be as effective (or more effective) as women quotas in increasing gender equality, while preventing the negative outcomes associated with quotas (Madison, 2017, 2019). For detailed reviews of the advantageous and disadvantageous outcomes of women quotas see Leszczyńska (2018) and Morgenroth and Ryan (2018).

To the best of our knowledge, there is no scientific research on the impact of men quotas in women-dominated professions or academic disciplines. Previous research has linked the influx of men into once female-dominated professions (e.g., computer programming, nurse anesthesia) to an elevation of status of the respective professions (Lindsay, 2007; Ensmenger, 2010). However, increasing the status of a female-typed profession does not necessarily help women. The example of computer programming – in its infancy a female-typed profession – demonstrates that increasing the social status of a profession can be associated with the marginalization of women in this profession (Ensmenger, 2010). Moreover, research suggests that the positive outcomes of increasing the number of men in female-typed professions are limited to men who receive higher pay compared to women (Budig, 2002; Lewis, 2018). In addition, in interview studies, men in female-dominated professions reported having some advantages that helped them progress more effectively up the career ladder than their female colleagues (Williams, 1992, 2013; Simpson, 2004). Thus, men quotas may change the status quo regarding the numerical representation of men in currently female-dominated professions. However, they may also enforce men’s higher social status by extending it to these professions.

### Predicting Perceptions of Women Quotas vs. Men Quotas

Unlike many other interventions designed to promote women in academia (e.g., special training, mentoring) that use a *fix the women* approach, women quotas represent a *fix the system* approach, that is, women quotas aim to change universities at an organizational level (Burkinshaw and White, 2017). This may make attitudes toward women quotas susceptible to system justifying motives (Costa-Lopes et al., 2013). *System justification* is the justification and rationalization of existing inequalities (e.g., favoring high-status groups over low-status groups; Jost and Banaji, 1994). System justification theory (Jost, 2011; Jost and van der Toorn, 2012) proposes that the status quo is not imposed by high-status groups (e.g., men) over low-status groups (e.g., women). In a collaborative effort, most individuals – including members of low-status groups – justify and rationalize existing inequalities. Maintaining the way things are, individuals regard existing social arrangements as fair and legitimate, even though these arrangements may do psychological and material harm to low-status individuals and groups (Jost and Hunyady, 2003, 2005).

The justification of inequalities appears paradoxical, particularly when low-status groups (e.g., women) defend the status quo against their self-interests. First, neither low- nor high-status individuals always justify the status quo (Brandt, 2013; Zimmerman and Reyna, 2013). However, individuals may justify the status quo in some situations, because perceiving the social context as stable, meaningful, and fair can prevent stress and help coping with stress, by fostering a sense of hope and control (Jost and Hunyady, 2003, 2005). For example, among women, system justification increased the perception of control over future outcomes, which was in turn associated with high self-esteem and physical health; among men, system justification was directly associated with self-esteem and physical health (McCoy et al., 2013). Unlike self-interest, system justification appears to be a more automated and implicit social motive; it has hence been linked to spontaneous, rather than deliberate perceptions (Jost and Banaji, 1994; Jost et al., 2004).

Among women and men, system justification predicted the rejection of feminism (Yeung et al., 2014) and opposition to different affirmative action policies for women (Phelan and Rudman, 2011; Fraser et al., 2015). Regarding the spontaneous perceptions of women and men quotas in academia, system justification may manifest as a “support paradox” (Van den Brink and Stobbe, 2014, p. 163). In interviews with policy makers, university professors, post-doctoral researchers, and Ph.D. students (most interviewees were women), Van den Brink and Stobbe (2014) found that in male domains, such as physics, any support given to women was regarded with suspicion, whereas support given to men was perceived as more natural and legitimate.

Research which specifically addresses reactions to women quotas for men-dominated professions is scarce, and research on reactions to men quotas for women-dominated professions is, to our best knowledge, non-existent. The objective of the present study is to investigate whether – in line with a support paradox – students, spontaneously, object more to women quotas than men quotas in academia. Specifically, we tested two hypotheses:

Hypothesis 1: Female and male students produce more negative free verbal associations with women quotas than men quotas in academia.
Hypothesis 2: Female and male students produce more emotional free verbal associations with women quotas than men quotas in academia.

In addition, we explored the semantic content of students’ free verbal associations to identify specific concerns with each quota.

## Method

### Sample

Data were obtained from a population of undergraduate students enrolled at the Medical University of Vienna, Austria. This population was chosen for several reasons. First, the Medical University of Vienna applies women quotas when hiring research and teaching personnel (Austrian University Act 120/2002 § 20b, 2002) and sometimes also when admitting students (Schmidt-Vierthaler, 2012; Winkler-Hermaden, 2012). Thus, the student population was both familiar with, and likely affected by, women quotas, either as the students of teachers who have been subject to quotas or as applicants to study at the university. Second, these students should also be familiar with the concept of men quotas in academia, because they have been discussed in Austria (Die Presse, 2014). Third, medicine includes both men-dominated (e.g., surgery) and women-dominated (e.g., pediatrics) sub-disciplines, which readily provides real-life exemplars to which both types of quotas can be applied.

Three hundred sixty-three students completed the study. Participants were excluded from data analyses for not indicating their gender, a central variable in this study (*n* = 23) and for being aged 35 and above, as senior students may differ from the general student population in unpredictable ways (*n* = 13). See Supplementary Material 1 for basic analyses of the responses of the cases excluded from the main analyses. The final sample included 327 students^3^ who, following a between-subjects design, either wrote down free associations to women quotas (*n* = 188) or men quotas (*n* = 139^4^). In both these subsamples, the proportions of women/men who responded were 49/51 percent. In addition, the two subsamples did not significantly differ in age or study progress^5^. Table 1 summarizes the sociodemographic information of the two subsamples.

**TABLE 1:** Participants’ sociodemographic characteristics by stimulus (women vs. men quotas).

	**Women quotas N (%)**	**Men quotas N (%)**
	
**Total**	**188 (100)**	**139 (100)**
**Gender**		
Women	92(48.9)	68(48.9)
Men	96(51.1)	71(51.1)
**Age**		
Younger than 20 years	11(5.9)	11(7.9)
20–25 years	132(70.2)	93(66.9)
26–30 years	36(19.1)	25(18.0)
31–35 years	9(4.8)	10(7.2)
**Study progress**		
1st year	30(16.9)	23(16.5)
2nd year	34(18.1)	24(17.3)
3rd year	31(16.5)	16(11.5)
4th year	27(14.4)	19(13.7)
5th year	24(12.8)	17(12.2)
6th year	29(15.4)	28(20.1)
7th year and above	12(6.4)	11(7.9)

### Procedure

The present study used a between-subject design with students either responding to women quotas or men quotas. Although a within-subjects design would have allowed more conclusive insights in the similarities and differences of students’ perceptions of both quotas, the associations with the second stimulus would not have been free and spontaneous, but inevitably influenced by the first stimulus.

The data were collected online using SoSci survey (Leiner, 2016). Email invitations containing the link and the password to the questionnaire were sent out via the university’s student office and one reminder was sent out after 4 weeks. The email invitation informed students that the study was about academic recruitment decisions. Upon clicking on the provided link, participants saw a consent form including basic information about the study (i.e., duration, participants’ rights, names and contact details of the responsible researchers). Upon giving consent, participants were randomly assigned to either produce free verbal associations with women quotas or men quotas. Participants completed the free association task at the beginning of the questionnaire. The stimuli *women quotas* and *men quotas* were mentioned first in the free association task. Then, participants provided sociodemographic information (i.e., gender, age, study progress). All research material was presented in German.

### Materials and Measures From the Free Association Task

To obtain free verbal associations to the quota concepts, participants were presented with a stimulus phrase and asked to write down every association that comes spontaneously to their minds. Participants were either presented with a stimulus phrase on women quotas (German: *Frauenquoten*) or a stimulus phrase on men quotas (German: *Männerquoten*). To frame the stimuli realistically, we adapted a phrase used in job ads by the Medical University of Vienna to communicate the application of quotas: “*To increase the proportion of women (men) in certain academic fields, some universities apply women (men) quotas in recruitment decisions. What do you associate with such women (men) quotas? Please list everything that you can think of spontaneously.”*^6^ For every association, participants had one separate line and two lines were displayed at the start screen. Upon entering an association, one additional line appeared to encourage further associations. Participants had to give a minimum of one association to complete the study and could list a maximum of ten associations. Upon completing the association task, participants saw a summary of their associations and were asked to rate the valence (positive, neutral, negative) of each association. Then, participants had to indicate for each association whether its content had emotional relevance for them (emotional/not emotional). Supplementary Material 2 illustrates the free associations task.

The following measures were available for analyses. A case number was assigned to each participant to determine how many and which free verbal associations were produced by each participant. Note that participants could produce between one and 10 free associations and that therefore, the number of associations per participant varied. The free verbal associations provided quantitative measures and qualitative information for analyses. The quantitative measures included the valence of each free association on a three-point ordinal scale (positive = 1, neutral = 2, negative = 3) and the emotionality of each free association on a binary scale (emotional = 1, not emotional = 0). The qualitative information was the semantic content of the free verbal associations (e.g., fair, unfair, necessary, unnecessary).

## Results

Overall, we counted 553 associations with the stimulus *women quotas*, of which 204 (36.9%) were different words, and 372 associations for the stimulus *men quotas*, of which 162 (43.5%) were different words. On average, women produced 2.87 (*SD* = 1.42), and men 3.01 (*SD* = 1.41) associations with women quotas. With men quotas, women produced 2.76 (*SD* = 1.36) and men 2.59 (*SD* = 1.21) associations. Results from a Poisson regression analysis revealed that neither quota, *p* = 0.696, nor gender, *p* = 0.574, nor quota × gender, *p* = 0.401, had a significant effect on the mean counts of free associations.

Of all associations with women quotas, one (0.2%) included the concept of men quotas, that is, the term “men quotas” was mentioned. Of all associations with men quotas, 31 (8.3%) included the concept of women quotas, that is, the term “women quotas” was mentioned. To avoid confounding effects as much as possible, these associations were removed from subsequent analyses.

### Hypothesis 1: Female and Male Students Produce More Negative Free Verbal Associations With Women Quotas Than Men Quotas in Academia

Overall, students’ free verbal associations with women quotas and men quotas were mostly negative, followed by neutral associations. The frequencies, proportions, and odds of positive, neutral, and negative associations by quota and gender are provided in Table 2. To determine whether the valence of the free associations differed significantly by quota, gender, or quota × gender, we performed a multilevel ordinal logistic regression analysis in R using the *ordinal* package (Christensen, 2019). The regression model included a random intercept for participants to control for individual differences among them and the fixed parameters – quota, gender, and quota × gender. Comparing this model to a baseline model that included only the intercept (see Field et al., 2012) revealed that it was overall significant from the baseline model as indicated by a statistically significant difference in the log likelihood, Δ*−2LL* = 47.11, *p* < 0.0001. Examining the individual predictors revealed that quota had a significant negative effect on the valence of the free associations, *b* = −0.65, *z* = −2.31, *p* = 0.021, indicating that the free associations with women quotas were more negative compared to the free associations with men quotas. The effects of gender, *p* = 0.548, and quota × gender, *p* = 0.107, were not statistically significant. Moreover, only a small amount of variance in the valence of the free associations was explained by individual differences among participants, σ*^2^* = 1.19, *SD* = 1.09. In sum, these results confirmed Hypothesis 1. Students’ free associations with women quotas were significantly more negative than their free associations with men quotas. See Table 3 for a summary of the results.

**TABLE 2:** Frequencies, proportions, odds, and odds ratios of the valence and the emotionality of the free associations by quota and gender.

	**Valence**			**Emotionality**	
	**Positive**	**Neutral**	**Negative**	**Emotional**	**Not emotional**
**Women quotas**					
Women					
Frequency	43	51	168	81	183
Proportion	0.16	0.20	0.64	0.31	0.69
Odds	0.20	0.24	1.79	0.44	2.26
Men					
Frequency	57	50	180	117	170
Proportion	0.20	0.17	0.63	0.41	0.59
Odds	0.25	0.21	1.68	0.69	1.45
Odds Ratios (Women/Men)	0.80	1.14	1.07	0.64	1.56
**Men quotas**					
Women					
Frequency	36	51	85	44	128
Proportion	0.21	0.30	0.49	0.26	0.74
Odds	0.27	0.42	0.98	0.35	2.91
Men					
Frequency	27	40	102	53	116
Proportion	0.16	0.24	0.60	0.31	0.69
Odds	0.19	0.31	1.52	0.46	2.19
Odds ratios (Women/Men)	1.42	1.36	0.65	0.76	1.30
**Odds ratios (women quotas/men quotas)**					
Women	0.74	0.57	1.83	1.26	0.78
Men	1.32	0.68	1.11	1.50	0.66

*The total numbers of associations with women quotas are N = 264 among women and N = 287 among men. The total numbers of associations with men quotas are N = 172 among women and N = 169 among men.*

**TABLE 3:** Multilevel ordinal logistic regression on the valence of the free verbal associations.

	***b***	***SE***	***z***	***p***
Intercept 1| 2	–2.05	0.21	–9.76	< 0.0001
Intercept 2| 3	–0.72	0.19	–3.81	< 0.0001
Quota	–0.65	0.28	–2.31	0.021
Gender	–0.15	0.26	–0.60	0.548
Quota × gender	0.64	0.40	1.61	0.107

Random effect	σ^2^ = 1.19			
	*SD* = 1.09			

*Baseline -2LL = 1,685.88, Model -2LL = 1,638.78, Δ-2LL = 47.10, p < 0.0001. Codes for DV: positive = 1, neutral = 2, negative = 3. Dummy codes for fixed parameters, Quota: Women quota = 0, Men quota = 1, Gender: Women = 0, Men = 1. This analysis was based on 889 associations, and 326 research participants.*

### Hypothesis 2: Female and Male Students Produce More Emotional Free Verbal Associations With Women Quotas Than Men Quotas in Academia

Students indicated that most of their free verbal associations were not emotional. The frequencies, proportions, and odds of emotional and non-emotional associations by quota and gender are displayed in Table 2. To investigate whether the odds of producing emotional vs. non-emotional associations differed significantly by quota, gender, or quota × gender, we performed a multilevel binomial logistic regression analysis in R using the packages *lme4* (Bates et al., 2015) and *lmtest* (Hothorn et al., 2019). Again, a random intercept for participants was included to control for individual difference among them. In addition, the fixed parameters quota, gender, and quota × gender were included in the model. Comparing this model to the intercept-only baseline model (Field et al., 2012) revealed that it was overall significant from the baseline model, as indicated by a statistically significant difference in the log likelihood, Δ*-2LL* = 328.98, *p* < 0.0001. However, examining the individual predictors revealed that neither quota, *p* = 0.886, nor gender, *p* = 0.240, nor quota × gender, *p* = 0.585, had a significant effect on the emotionality of the free associations. Instead, the analysis showed that a large amount of variance in the odds of emotional vs. non-emotional associations was explained by individual differences among the participants, σ*^2^* = 120.60, *SD* = 10.98. In sum, these results did not confirm Hypothesis 2. The odds to indicate one’s associations as emotional vs. not emotional were not influenced by quota, gender, or quota × gender. Instead, they varied greatly based on individual differences among the students. See Table 4 for a summary of the results.

**TABLE 4:** Multilevel binomial logistic regression on the odds of emotional vs. not emotional associations.

	***b***	***SE***	***Z***	***p***
Intercept	–7.66	0.88	–8.67	< 0.0001
Quota	–0.15	1.05	–0.14	0.886
Gender	1.17	1.00	1.18	0.240
Quota × gender	–0.81	1.49	–0.055	0.585

Random effect	σ^2^ = 120.60			
	*SD* = 10.98			

*Baseline -2LL = 1,131.48, Model -2LL = 802.50, Δ-2LL = 328.98, p < 0.0001. Codes for DV: emotional = 1, not emotional = 0. Dummy codes for fixed parameters: Quota: Women quota = 0, Men quota = 1, Gender: Women = 0, Men = 1. This analysis was based on 891 associations and 326 research participants.*

### Exploration of the Sematic Content

The free associations were first categorized in order to analyze their semantic content. An independent rater who was naïve to the research questions inductively created 16 categories based on the associations’ content. Then, two independent and naïve raters (one woman, one man) assigned the associations to the categories. The inter-rater agreement was substantial, *Cohen’s Kappa* = 0.72. Sorted by size, listing the largest category first, the categories were:

1. “Unfair” (*n* = 138), that is, perceptions that quotas decrease fairness and discriminate against the other gender (e.g., unfair, discriminating).
2. “Gender controversy” (*n* = 97), that is, references to gender issues (e.g., gender, feminism, sexism).
3. “Counterproductive” (*n* = 89), that is, the perception that quotas are not a viable solution to achieve gender equality (e.g., wrong solution, enforced).
4. “Derogatory” (*n* = 83), that is, concerns that beneficiaries of quotas may be seen negatively by others (e.g., derogatory, token women).
5. “Necessary” (*n* = 69), that is, perceptions that quotas are inevitable to achieve gender equality (e.g., necessary, chance).
6. “Fair” (*n* = 66), that is, perceptions that quotas increase fairness and promote gender equality (e.g., gender equality, fairness).
7. “Nonsensical” (*n* = 62), that is, expressions of a general, strong opposition to any quota (e.g., non-sense, I oppose quotas).
8. “Qualification vs. gender” (*n* = 58), that is, the perception that qualification should be the principal criteria in recruitment decisions (e.g., only qualification should count).
9. “Unnecessary” (*n* = 58), that is, perceptions that quotas are not needed, redundant, and/or exaggerated (e.g., unnecessary, exaggerated).
10. “Beneficial” (*n* = 45), that is, expressions of a general endorsement of quotas (e.g., balance, good, important).
11. “Politics” (*n* = 44), that is, associations referring to the representation of women and men in politics (e.g., representation, party).
12. “Leadership” (*n* = 25), that is, associations with economic leadership (e.g., leadership, corporate boards).
13. “Unknown” (*n* = 13), that is, expressions that quotas are unfamiliar (e.g., unfamiliar, not heard of it).
14. “Ambivalence” (*n* = 9), that is, expressions of ambivalence toward quotas (e.g., ambivalent, perhaps).
15. “Misandrist” (*n* = 4), that is, the impression that the aim of quotas is to hurt men (e.g., men hating).
16. “Hostility against women” (*n* = 4), that is, blatantly hostile comments about women (e.g., men are more capable than women).

One additional category (“Other”) included associations that could not be sorted into any of the categories (*n* = 26). In the subsequent analyses, we only considered categories of substantial size, that is, categories 1–12 that included at least 25 associations. Table 5 provides an overview of these categories and sample words for each category, and it shows the absolute frequencies assigned to the categories by quota and gender.

**TABLE 5:** Absolute frequencies of free verbal associations assigned to the categories by quota and gender.

		**Women quotas**		**Men quotas**	
**Category**	**Examples**	**Women *N* = 262**	**Men *N* = 287**	**Women *N* = 172**	**Men *N* = 169**
Beneficial	Balance, good, important	14	12	14	5
Counterproductive	Wrong solution, forced	28	39	11	11
Derogatory	Derogatory, token woman	31	37	7	8
Fair	Gender equality, fairness	17	15	25	9
Gender controversy	Gender, feminism, sexism	26	24	26	21
Leadership	Leadership, corporate board	5	6	11	3
Necessary	Necessary, chance	32	22	8	7
Nonsensical	Non-sense, I oppose quotas	11	16	10	25
Politics	Percentage, party	10	16	7	11
Qualification vs. gender	Only qualification should count	17	21	10	10
Unfair	Unfair, discriminating	44	51	21	22
Unnecessary	Unnecessary, exaggerated	21	14	4	19
Excluded from analysis		6	14	18	18

*The categories “Ambivalence,” “Misandrist,” and “Hostility against women” were excluded from MCA based on too few observations (N < 25). The category “other” was also excluded from MCA.*

To examine the semantic content of the free verbal associations with women quotas and men quotas, we performed a multiple correspondence analysis that, broadly, is a principal component analysis for qualitative data (Greenacre and Blasius, 2006; Abdi and Valentin, 2007). The analysis reduces the complexity of the data and uncovers underlying dimensions (Greenacre and Blasius, 2006). We performed multiple correspondence analysis using the R packages FactoMineR (Lê et al., 2008). We included the variables quota (women quotas, men quotas), participants’ gender (women, men), valence (positive, neutral, negative), emotionality (emotional, not emotional) and the categories described above (“unfair,” “gender controversy,” etc.) in the analysis. The results revealed that the two principal dimensions together explained 60.5% of variance in the data.

Dimension 1 explained 47.3% of variance in the data. Breaking down the contribution of each variable to Dimension 1 revealed that negative valence, followed by neutral valence, explained the largest proportions of its variance. Additional variables explaining more than five percent of the variance of Dimension 1 were women quotas, men quotas, positive valence and the categories “beneficial,” “counterproductive,” “derogatory,” “fair,” and “unfair.” To examine the positions of these variables on Dimension 1, we computed correlation coefficients. We observed negative correlations of Dimension 1 to women quotas, negative valence, and the categories “counterproductive,” “derogatory,” and “unfair.” In addition, we observed positive correlations of Dimension 1 to men quotas, neutral and positive valence, and the categories “beneficial” and “fair.”

Dimension 2 explained 13.2% of variance in the data. Breaking down the contribution of each variable to Dimension 2 showed that positive valence explained the largest proportion of its variance. Additional variables that explained more than five percent of variance of Dimension 2 included women quotas, men quotas, emotional associations, and the categories “derogatory,” “fair,” “necessary,” and “non-sensical” Again, we computed correlation coefficients to examine the positions of each variable on Dimension 2. We observed negative correlations of Dimension 2 to men quotas and the category “nonsensical,” and positive correlations of Dimension 2 to women quotas, emotional associations, and the categories “derogatory,” “fair,” and “necessary.” Table 6 summarizes the contribution of variance of each variable to Dimension 1 and Dimension 2, as well as their correlations to the two dimensions.

**TABLE 6:** Description of the two dimensions extracted in the multiple correspondence analysis.

	**Dimension 1**		**Dimension 2**	
**Adjusted inertia in %**	**47.4**		**13.2**	
	**σ^2^**	***r***	**σ^2^**	***r***
**Quota**				
Women quota	**5.39**	–0.26	**6.34**	0.24
Men quota	**9.32**	0.26	**10.97**	–0.24
**Gender**				
Women	4.61	0.20	0.16	−
Men	4.39	–0.20	0.15	−
**Valence**				
Positive	**8.85**	0.25	**22.23**	0.52
Neutral	**10.08**	0.25	3.44	–0.32
Negative	**12.00**	–0.50	2.18	–0.20
**Emotionality**				
Emotional	2.87	–0.14	**7.07**	0.19
Not emotional	1.45	0.14	3.58	–0.19
**Category**				
Beneficial	**5.97**	0.60	3.23	0.42
Counterproductive	**5.11**	–0.55	0.34	–0.11
Derogatory	**7.20**	–0.65	**10.31**	0.55
Fair	**9.13**	0.61	**6.89**	0.51
Gender controversy	3.43	0.26	2.86	–0.29
Leadership	2.64	0.52	1.03	–0.34
Necessary	0.92	0.12	**7.05**	0.50
Nonsensical	0.19	−	**5.16**	–0.47
Politics	0.14	−	0.01	−
Qualification vs. gender	0.01	−	0.37	–0.14
Unfair	**5.46**	–0.47	3.31	–0.26
Unnecessary	0.85	–0.33	3.33	–0.39

*σ^2^ = contribution of variance of each variable to the dimension. Variables that explain more than 5 percent of the variance are displayed in bold. r = correlation of each variable with the dimension. Only statistically significant correlation coefficients are displayed.”*

In sum, these results indicate that most variance in the data was explained by the dichotomy between negative views of women quotas, that is, as counterproductive, unfair, and derogatory, and neutral and positive views of men quotas, that is, as beneficial and fair (Dimension 1). In addition, a small amount of variance was explained by the dichotomy between perceiving women quotas as emotional, necessary, fair, albeit derogatory, and perceiving men quotas as being nonsensical (Dimension 2).

**FIGURE 1:**
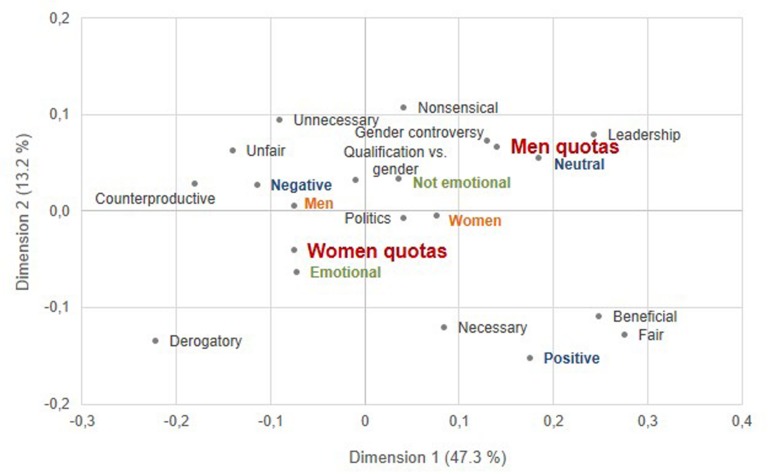
Biplot of the multiple correspondence analysis with the stimuli women quotas and men quotas (red), participant gender (orange), association valence (blue), association emotionality (green), and association category (gray). The biplot is interpreted by examining the position of the variables (i.e., gender, valence, emotionality) and the semantic content (i.e., the categories) along Dimension 1 and 2 (Greenacre and Blasius, 2006). In addition, the spatial distance between the variables and the semantic content reflects their relationships to each other. The closeness of points to each other represents the frequency of connections in the data (Abdi and Valentin, 2007).

Figure 1 visualizes these results in one plot. The plot shows the semantic room along the two principal dimensions. It is interpreted by examining the position of the nominal variables (i.e., gender, valence, emotionality) and the semantic content (i.e., the categories) along the two dimensions (Greenacre and Blasius, 2006). In addition, the spatial distance between the nominal variables and the semantic content reflects the relationships of the variables to each other. The closeness of points to each other represents the frequency of connections in the data (Abdi and Valentin, 2007). A visual analysis of Figure 1 confirmed that on Dimension 1, women quotas were positioned close to the variable “negative” and the categories “counterproductive,” “derogatory,” and “unfair,” whereas men quotas were positioned close to the variables “neutral” and “positive” as well as to the categories “beneficial” and “fair.” On Dimension 2, women quotas were positioned close to the variables “positive” and “emotional,” indicating that positive associations with women quotas were frequently rated as emotional. Moreover, on Dimension 2, women quotas were positioned close to the categories “beneficial” and “necessary,” whereas men quotas were positioned close to the category “nonsensical”. In addition, Figure 1 shows that the category “derogatory” was positioned closer to women quotas than to men quotas. This indicates that the derogation of quota beneficiaries was more frequently associated with women quotas than men quotas. In addition, the category “derogatory” was positioned close to the variable “emotional”, which means that this category was frequently indicated as being emotional.

## Discussion

We investigated whether students spontaneously perceived the preferential treatment of women more negatively and more emotionally than the preferential treatment of men. In sum, we found that female and male students produced more negative associations with women quotas than with men quotas in academia. In contrast to our predictions, women quotas were not perceived as more emotional than men quotas. In addition, examining the semantic content of the free verbal associations revealed that concerns over negative perceptions of beneficiaries (e.g., as less competent) were more frequently associated with women quotas than men quotas.

The main limitation of the present study are the ordinal and binary response formats for the associations’ valence and emotionality ratings. In both cases, more finely graduated rating scales would have increased the study’s statistical power. Another limitation is that fewer students responded to men quotas than women quotas. More equal sample sizes would have increased the study’s power. Future studies could also benefit from a design that provides clear definitions of women and men quotas and examines participants’ reactions to those. Moreover, future studies could include system justification as an individual difference variable to more directly assess its association with perceptions of women and men quotas. Like this, future research could also test whether other individual difference variables (e.g., perceptions of anti-female and anti-male discrimination, perceptions regarding the competence of women and men) are better predictors of the perceptions of women and men quotas.

Confirming Hypothesis 1, both female and male students perceived women quotas more negatively than men quotas. To some extent, this may reflect a public debate where women quotas have been discussed controversially (He and Kaplan, 2017; Debating Europe, 2018), while men quotas were discussed considerably less. Accordingly, students who wrote down associations with women quotas did not mention men quotas, whereas students who produced associations with men quotas thought of women quotas.

Overall, the present results are inconsistent with previous explanations for the rejection of women quotas, such as that the *modus operandi* of quotas (i.e., preferential treatment) is primarily perceived to be unfair (Bobocel et al., 1998). In this case, perceptions of women quotas and men quotas should have been equally negative, neutral, or positive. Moreover, the present results are inconsistent with the idea that only being a beneficiary, or not, affects reactions to quotas (Kravitz and Platania, 1993; Konrad and Hartmann, 2001). Had this been the case, women would have perceived women quotas more favorably than men quotas, whereas men would have perceived men quotas more favorably than women quotas.

The present results are in line with our predictions based on system justification theory, according to which individuals have a tendency to perceive the status quo as natural, fair, and legitimate (Jost and Kay, 2005; Jost et al., 2010). Moreover, our results are consistent with research showing that support for men was often perceived as more natural and legitimate than support for women (Van den Brink and Stobbe, 2014). The analysis of the semantic content further supported the quantitative results. Both female and male students perceived women quotas as counterproductive, unfair, and derogatory, whereas men quotas were perceived as beneficial and fair. However, system justification has been linked to automated and implicit, rather than deliberate perceptions (Jost and Banaji, 1994; Jost et al., 2004). Assessing free associations, which are thought to measure default thinking (Rozin et al., 2002; Joffe and Elsey, 2014), we also tapped into students’ spontaneous thoughts and perceptions of women and men quotas. Asking students more directly about their support for quotas in academia might have produced different results, which might have been more consistent with other explanations of reactions to quotas, such as self-interest (Kravitz and Platania, 1993; Konrad and Hartmann, 2001).

Rejecting Hypothesis 2, we found that women quotas were not perceived more emotionally than men quotas. Instead, the random effect of participants explained a substantial proportion of variance in the emotionality of associations. The analysis of the free verbal associations’ semantic content uncovered which variables were deemed emotional by the students. Overall, women quotas were more frequently linked to emotional associations. In particular, the positive associations with women quotas were emotional. In addition, the category “derogatory” was associated with emotionality. Thus, concerns over negative perceptions of quota beneficiaries (e.g., as less competent) appear to elicit emotions.

Lastly, we found that negative perceptions of beneficiaries were a specific concern about women quotas. This finding is inconsistent with previous explanations for the so-called “stigma of incompetence,” that is, an attributional bias (Heilman et al., 1992, p. 537). As individuals seek to find causes for the success of others, they tend to overestimate external factors and underestimate internal factors (see attribution theory, Kelley, 1973). Thus, in the presence of preferential treatment (i.e., an external factor), women’s competence (i.e., an internal factor) was discounted (Heilman, 1996; Resendez, 2002; Heilman and Welle, 2006). However, following this logic, all quota beneficiaries, including men, should be perceived negatively.

Most research has only studied the effects of preferential treatment on the perception of female beneficiaries (Heilman et al., 1992; Resendez, 2002; Heilman and Welle, 2006), or other minority beneficiaries, such as African Americans (Maio and Esses, 1998; Evans, 2003). However, it seems plausible that not all beneficiaries of preferential treatment are perceived negatively. Perhaps, male quota beneficiaries would be protected from negative perceptions because masculine attributes are associated with higher social status (Rudman et al., 2012). After all, high social status and perceived competence are often confounded and gender stereotypes typically ascribe competence more to men than women (Fiske et al., 2002; Ellemers, 2018; Fiske, 2018). However, recent research showed that today, women are ascribed with equally as much or more competence than men (Eagly et al., 2020). Alternatively, negative perceptions of female quota beneficiaries may also present a way to penalize women for entering men-dominated, high-status professions. Experimental research showed that women who were described as successful in male domains were perceived negatively and disliked (Heilman et al., 2004; Heilman and Okimoto, 2007), and women who sought high-status positions faced backlash in the form of dislike and personal derogation (Rudman and Glick, 2001; Rudman et al., 2012). Similarly, research revealed that quota women were perceived as threatening to men; and the more threatened men felt the more they perceived quota women in stereotypical female terms (Faniko et al., 2017).

The present findings are consistent with the notion of a support paradox, that is, in men-dominated domains, support for men is perceived as fairer and more legitimate than support for women (Van den Brink and Stobbe, 2014). System justification, that is, the rationalization of existing inequalities such as favoring high-status groups (i.e., men) over low-status groups (i.e., women) may underlie the support paradox (Jost, 2011; Jost and van der Toorn, 2012). System justification seems paradoxical, and at least among women, at odds with their self-interests. However, when it comes to quotas, self-interest may be more complex than simply being a beneficiary of the policy or not. System justification may be in the self-interest of low-status groups. For example, research found that women with system justifying beliefs (i.e., who rationalize inequalities) perceived greater control over future outcomes, which in turn was positively associated with self-esteem and physical health (McCoy et al., 2013). In particular, among young women who are at the beginning of their professional careers, maintaining a belief that the world is a fair place and that pursuing an education will be rewarded may be an important strategy helping them to stay motivated during their studies (e.g., Fishman and Husman, 2017).

Perceiving the academic system as fair and legitimate may also have advantages for male students. They may benefit from trusting that any opportunity they received was deserved and not based on external help in the form of a pro-male bias. Research showed that women’s self-esteem was negatively affected by benefitting from external help (i.e., preferential treatment; Heilman, 1996; Unzueta et al., 2010; Leslie et al., 2014). Men’s self-esteem was found to benefit from believing that others received more help (i.e., through a rigid quota) than they actually did (Unzueta et al., 2008). However, to our best knowledge, no studies have directly examined the effects of external help for men (e.g., through pro-male bias) on their self-esteem and self-perceived competence.

Apart from system justification, there may be additional explanations for the present results. First, as women quotas are much more common than men quotas, there may be more instances where women quotas have challenged students’ fairness perceptions (e.g., when women quotas were used in student admittance to the Medical University of Vienna; Schmidt-Vierthaler, 2012; Winkler-Hermaden, 2012). Second, men-dominated domains are commonly ascribed higher status than women-dominated domains (England, 2010; Block et al., 2018). Thus, women quotas that help a low-status group gain access to a high-status domain may be perceived as a greater breach to meritocracy compared to men quotas that favor a high-status group for low-status domains. Third, students may generally perceive men to be more competent academics than women, and thus, perceive that women quotas breach meritocracy more than men quotas. Previous research showed that across culture, science was implicitly associated more with men than women (Nosek et al., 2009). Science-is-male associations have also been found among scientists and students (Smyth and Nosek, 2015).

Fourth, female and male students may not perceive structural discrimination against women and therefore, may perceive women quotas as an unfair advantage for women. Previous research suggests that university students often do not perceive gender discrimination and expect to enter a gender-neutral workplace (Sipe et al., 2009). In addition, students’ perceptions of men quotas as fair may be associated with perceptions of discrimination against men. In previous research, male medical students reported more incidences of educational inequalities, such as favoritism of female students and bias toward male students compared to female medical students; male medical students felt particularly discriminated against in women-dominated domains (e.g., gynecology, obstetrics; Witte et al., 2006). In addition, students may perceive that women are generally favored over men in academia, a perception that women quotas may have contributed to. Previous research showed that perceptions of anti-male discrimination have been rising in recent years (Kehn and Ruthig, 2013; Ruthig et al., 2017). From this perspective, men quotas may be perceived as a beneficial and fair tool to counter anti-male discrimination.

## Conclusion

In sum the present findings indicate a support paradox regarding women quotas and men quotas in academia. Female and male students perceived women quotas as less fair than men quotas. Future research should study the impact of men quotas in women-dominated domains and further investigate the factors underlying reactions to men quotas.

## Data Availability Statement

The raw data supporting the conclusions of this article will be made available by the authors, without undue reservation, to any qualified researcher.

## Ethics Statement

The studies involving human participants were reviewed and approved by Inneruniversitäre Datenschutzkommission MedUni Wien (Internal data protection committee of the Medical University of Vienna). The patients/participants provided their written informed consent to participate in this study.

## Author Contributions

MZ developed the conception and design of the study, collected the data, and performed the statistical analyses. EK supervised the development of the study design and data analyses. MZ wrote the first draft of the manuscript. MZ and EK contributed to manuscript revision, read and approved the submitted version.

## Conflict of Interest

The authors declare that the research was conducted in the absence of any commercial or financial relationships that could be construed as a potential conflict of interest.

## Footnotes

1 In Austria, all federal institutions and organizations, including universities, are mandated to preferentially select a female candidate over an equally qualified male candidate for any position until a quota of at least 50 percent women holding equivalent positions is fulfilled (Austrian Federal Act for Gender Equality 100/1993 § 11b, 1993; Austrian University Act 120/2002 § 20b, 2002).

2 Commonly, policy makers, researchers, and journalists use the term “gender quotas” (Debating Europe, 2018; International Institute for Democracy and Electoral Assitance, 2018; Morgenroth and Ryan, 2018; European Institute for Gender Equality, 2019). Some have argued though that the term “sex quotas” would be more accurate (Madison, 2019). Indeed, it is unclear whether quotas are applied based on gender or sex assigned at birth. Considering this, we refer to “preferential treatment based on sex/gender”.

3 The final sample size (*n* = 327) included 4.7% of the total student population of the Medical University of Vienna at the time of data collection. To rule out response bias, we compared early-respondents (after first email invitation) with late-respondents (after second email invitation) by regressing quota, gender, and quota × gender on early vs. late response. The results from the binomial logistic regression analysis revealed that neither quota, *p* = 0.484, nor gender, *p* = 0.398, nor quota × gender, *p* = 0.201, predicted early compared to late response. The similarities in the responses of early and late respondents indicate that non-respondents also may not differ from respondents in meaningful ways (Bortz and Döring, 2006).

4 The lower number of participants associating with men quotas was based on a higher drop-out rate in this group, which we attributed to a lower familiarity with men quotas; the word “unknown” was associated with men quotas (*n* = 13) but not with women quotas.

5 To examine whether the two subsamples differed by participants’ gender, age, or study progress, we performed a binomial logistic regression analysis regressing these sociodemographic variables on the assignment to the two subsamples. Neither gender, *p* = 0.979, nor age, *p* = 0.979, nor study progress, *p* = 0.534, predicted the assignment to the subsample, indicating that there were no significant sociodemographic differences between participants producing free verbal associations with women and men quotas.

6 Note that the German term for quota can be ambiguous; it can refer to either a given percentage or to the preferential treatment of women and men. Thus, we used the phrase “to increase the number of women/men” to make sure that participants understood that we referred to the preferential treatment of women/men.
