# Supplementary material for: Women Quotas vs. Men Quotas in Academia: Students Perceive Favoring Women as Less Fair Than Favoring Men
Source: Front Psychol. 2020 Apr 28;11:700. doi: 10.3389/fpsyg.2020.00700 (PMC7198813; doi:10.3389/fpsyg.2020.00700)
Supplement: Supplementary file 1 [file Table_1.docx]

| Supplement 1.  Frequencies, proportions, odds, and odds ratios of the valence and the emotionality of the free associations of the excluded cases by quota | | | | | |
| --- | --- | --- | --- | --- | --- |
|  | Valence | | | Emotionality | |
|  | Positive | Neutral | Negative | Emotional | Not emotional |
| **Unknown gender** |  | | |  | |
| Women quotas |  | | |  | |
| Frequency | 10 | 6 | 14 | 8 | 22 |
| Proportion | .33 | .20 | .47 | .27 | .73 |
| Odds | .50 | .25 | .88 | .36 | 2.75 |
|  |  | | |  | |
| Men quotas |  | | |  | |
| Frequency | 3 | 8 | 18 | 17 | 12 |
| Proportion | .10 | .28 | .62 | .59 | .41 |
| Odds | .12 | .38 | 1.64 | 1.42 | .71 |
|  |  |  |  |  |  |
| Odds Ratios  (Women quotas/ Men quotas) | 4.17 | .66 | .54 | .25 | 3.87 |
|  |  |  |  |  |  |
| **Aged 35 to 60** |  |  |  |  |  |
| Women quotas |  | | |  | |
| Frequency | 16 | 1 | 11 | 8 | 20 |
| Proportion | .57 | .04 | .39 | .29 | .71 |
| Odds | 1.33 | .04 | .65 | .40 | 2.50 |
|  |  | | |  | |
| Men quotas |  | | |  | |
| Frequency | 4 | 1 | 2 | 2 | 5 |
| Proportion | .57 | .14 | .29 | .31 | .69 |
| Odds | 1.33 | .17 | .40 | .40 | 2.50 |
|  |  |  |  |  |  |
| Odds Ratios  (Women quotas/ Men quotas) | 1.00 | .24 | 1.63 | .76 | 1.30 |
| Note. The Ns for participants with unknown gender are 12 (Women quotas) and 11 (Men quotas). The N for participants classified as senior students are 10 (Women quotas) and 3 (Men quotas). | | | | | |

***Supplementary Material***

**Women Quotas vs. Men Quotas in Academia: Students Perceive Favoring Women as Less Fair Than Favoring Men**

Miriam K. Zehnter* & Erich Kirchler

*Correspondence: [miriam.zehnter@univie.ac.at](mailto:miriam.zehnter@univie.ac.at)
