# Supplementary material for: Women Quotas vs. Men Quotas in Academia: Students Perceive Favoring Women as Less Fair Than Favoring Men
Source: Front Psychol. 2020 Apr 28;11:700. doi: 10.3389/fpsyg.2020.00700 (PMC7198813; doi:10.3389/fpsyg.2020.00700)
Supplement: Supplementary file 2 [file Table_2.docx]

***Supplementary Material***

Women Quota vs. Men Quota in Academia: Students Perceive Favoring Women as Less Fair Than Favoring Men

Miriam K. Zehnter* & Erich Kirchler

*Correspondence: [miriam.zehnter@univie.ac.at](mailto:miriam.zehnter@univie.ac.at)

| Supplement 2  Illustration of the free association task on the example of “women quotas” | | | | | |
| --- | --- | --- | --- | --- | --- |
| **To increase the proportion of women in certain academic fields, some universities apply *women quotas* in recruitment decisions.**  What do you associate with such ***women quotas***? Please list everything that you can think of ***spontaneously***. | | | | | |
| *unfair* |  | | | | |
|  | | | | | |
| *counterproductive* |  | | | | |
|  |  | | | | |
| **Please look at your associations and indicate whether you consider them *positive*, *neutral*, or *negative*.** | | | | | |
|  | | positive | neutral | | negative |
| *unfair* | | O | O | | O |
| *counterproductive* | | O | O | | O |
|  | | | | | |
| **Please indicate for each association whether it is *emotional* for you.** | | | | | |
|  | | Yes | | No | |
| *unfair* | | O | | O | |
| *counterproductive* | | O | | O | |
|  | | | | | |
| Note: A minimum of one association was required; a maximum of 10 associations was possible. | | | | | |
